# Niosomes, an alternative for liposomal delivery

**DOI:** 10.1371/journal.pone.0194179

**Published:** 2018-04-12

**Authors:** Rianne Bartelds, Mohammad Hadi Nematollahi, Tjeerd Pols, Marc C. A. Stuart, Abbas Pardakhty, Gholamreza Asadikaram, Bert Poolman

**Affiliations:** 1 Department of Biochemistry, University of Groningen, Groningen, The Netherlands; 2 Department of Clinical Biochemistry, School of Medicine, Medical University Campus, Kerman, Iran; 3 Department of Electron Microscopy, University of Groningen, Groningen, The Netherlands; 4 Pharmaceutics Research Center, Institute of Neuropharmacology, Kerman University of Medical Science, Medical University Campus, Kerman, Iran; 5 Endocrinology and Metabolism Research Center, Institute of Basic and Clinical Physiology Sciences, Kerman University of Medical Sciences, Medical University Campus, Kerman, Iran; University of Waterloo, CANADA

## Abstract

Niosomes are used in studies for drug delivery or gene transfer. However, their physical properties and features relative to liposomes are not well documented. To characterize and more rationally optimize niosome formulations, the properties of these vesicle systems are compared to those of liposomes composed of phosphatidylcholine and phosphatidylethanolamine lipids plus cholesterol. Niosomes are highly stable and only slightly more leaky than liposomes as assayed by calcein leakage; the permeability for ions (KCl) is higher than that of liposomes. Contrary to liposomes, the size of niosomes decreases substantially upon freezing in liquid nitrogen and subsequent thawing, as shown by cryo-EM and dynamic light scattering. The packing of niosomal membranes was determined by laurdan fluorescence and is slightly lower than that of liposomes. We did not succeed in the functional reconstitution of the L-arginine/L-ornithine antiporter ArcD2 in niosomes, which we attribute to the non-ionic nature of the surfactants. The antimicrobial peptides alamethicin and melittin act similarly on niosomes and liposomes composed of unsaturated components, whereas both niosomes and liposomes are unaffected when saturated amphiphiles are used. In conclusion, in terms of stability and permeability for drug-size molecules niosomes are comparable to liposomes and they may offer an excellent, inexpensive alternative for delivery purposes.

## Introduction

**Fig 1:**
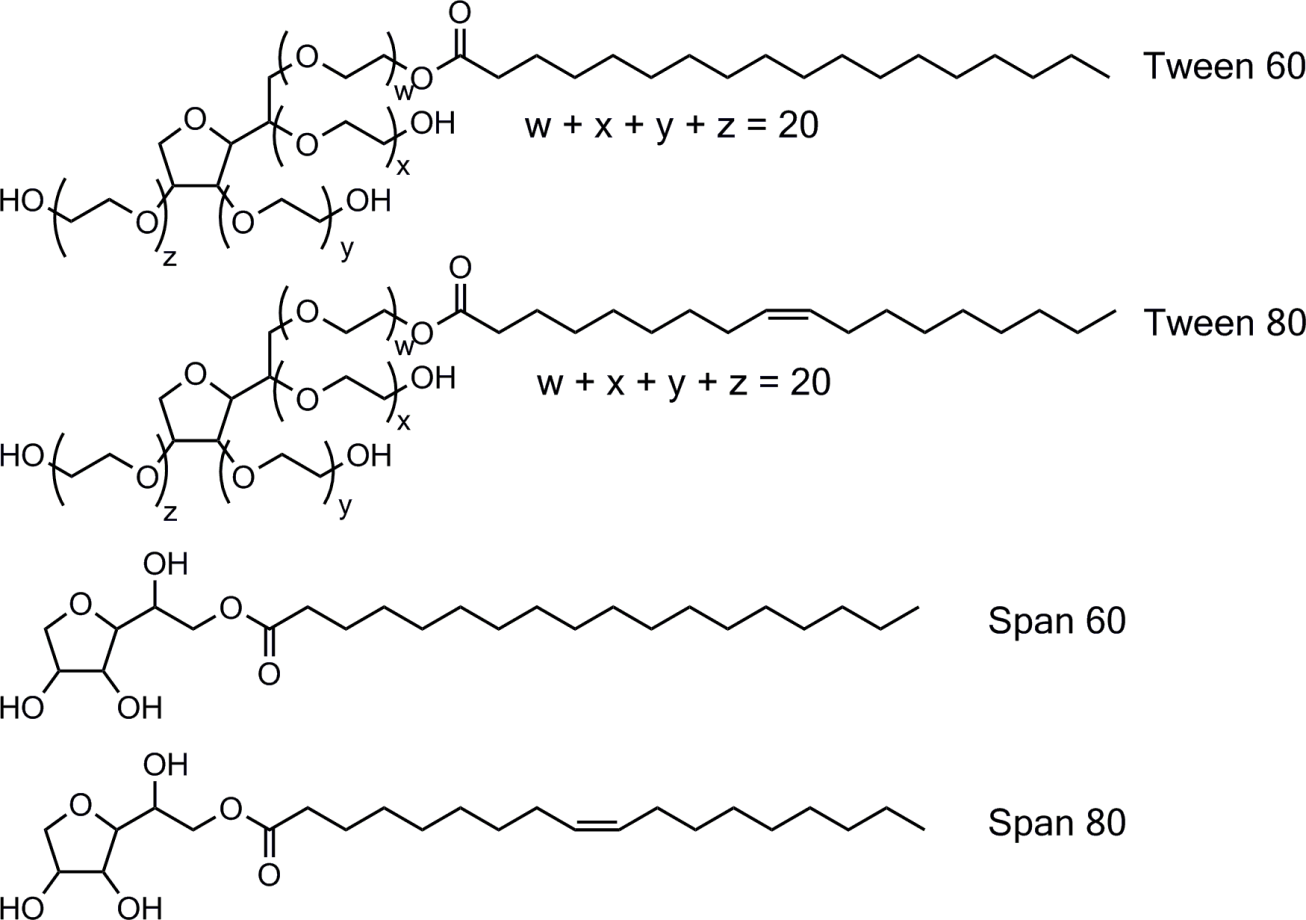
Examples of non-ionic surfactants used in this study.

Niosomes are vesicles composed of non-ionic surfactants, amphipathic compounds with an overall neutral charge (see Fig 1 for the structures of the surfactants used in this study). These non-ionic surfactants are cheap and safe for use in biomedicine, *e*.*g*. as niosomal drug carriers for both hydrophilic and hydrophobic drugs. The *in vitro* and *in vivo* effects of niosome-encapsulated drugs are reviewed in[1] and more recent studies by[2], but little is known about the biophysical properties of niosomes. Throughout this paper, vesicles (mainly) composed of non-ionic surfactants are called niosomes, while vesicles (mainly) composed of phospholipids are indicated as liposomes.

Niosomes have been shown to confine many compounds as measured by their entrapment efficiency. The entrapment efficiency of water-soluble dyes depends on the vesicle formation method, with the ether injection method giving a higher carboxyfluorescein entrapment than vesicle formation by lipid solvation via mild mechanical treatment or sonication[3]. Cholesterol was found to increase the entrapment efficiency of Span 60 and Span 80 niosomes[4, 5] but also of bolaform surfactants[6]. The stability of niosomes has been mostly referred from the time-dependent release of water-soluble dyes [3]. Kato and coworkers found that the stability of Span 80 niosomes is temperature-dependent. At 42°C, the niosomes fused, while at 4 and 25°C they formed stable structures. They reported that leakage of the water-soluble dyes brilliant blue FCF and indigo carmine from niosomes is greater than from liposomes[7]. Hayashi *et al*, found that the headgroups of Span 80 niosomes are more motile and less hydrophobic than those of zwitterionic lipids in liposomes[8]. This results in a higher water permeability compared to liposomes. In a follow-up study, it was shown that Span 80 vesicles perturb and hemifuse to phospholipid vesicles[9]. This is an important feature for delivery, since full fusion of niosomes with phospholipid vesicles transfers the niosomal content and is a first step for fusion to mammalian cells and delivery of drugs or genes in those cells. So far, fusion between niosomes and cells has not been reported. All these studies were carried out using Span 80 vesicles (which sometimes contained cholesterol and/or phospholipids), niosomes composed of other amphiphiles and mixtures of amphiphiles have been used for drug delivery (reviewed by[1, 2]). Cholesterol has been shown to diminish the leakage of carboxyfluorescein from niosomes[3].

To better understand the delivery properties and to improve the formulations used, we studied niosomes of different compositions and compared them to liposomes. We report the stability, permeability and membrane fluidity of niosomes, using vesicles composed of the saturated surfactants Tween 60, Span 60 and cholesterol or the unsaturated surfactants Tween 80, Span 80 and cholesterol, and compare the niosomes to liposomes composed of the saturated phospholipids DPPC, DPPE and cholesterol or the unsaturated phospholipids DOPC, DOPE and cholesterol. The results and use of niosomes as alternative in drug and gene delivery are discussed.

## Materials and methods

### Materials

Span 60, Span 80, Span 85, Tween 60, Tween 80, calcein, ETH-157, L-ornithine monohydrochloride and L-arginine were obtained from Sigma-Aldrich. 1,2-dipalmitoyl-*sn*-glycero-3-phosphocholine (DPPC), 1,2-dioleoyl-*sn*-glycero-3-phosphocholine (DOPC), 1,2-dipalmitoyl-*sn*-glycero-3-phosphoethanolamine (DPPE), 1,2-dioleoyl-*sn*-glycero-3-phosphoethanolamine (DOPE), 1,2-dioleoyl-*sn*-glycero-3-phosphoglycerol (DOPG), and cholesterol were purchased from Avanti Polar Lipids. Laurdan and 8-hydroxy-1,3,6-pyrenetrisulfonate (pyranine) were obtained from Thermo Fisher Scientific. Melittin and alamethicin were from Serva Electrophoresis and Santa Cruz Biotechnology, respectively, and obtained via Bio-Connect Life Sciences. Radiolabeled L-[^14^C(U)]-arginine was ordered from Moravek, Inc.

### Vesicle formation

Both niosomes and liposomes were formed from the corresponding amphiphiles by thin film hydration. The amphiphiles, lipids or non-ionic surfactants, dissolved in chloroform:methanol (9:1) were mixed and dried by rotary evaporation and subsequently hydrated in the appropriate buffer. Liposomes were flash-frozen in liquid nitrogen and thawed (5x) and stored in liquid N_2_, after which the multilamellar vesicles were extruded 15x through a 200 nm polycarbonate filter (Avestin). Unless indicated otherwise, niosomes were not frozen in liquid nitrogen prior to extrusion, since this reduces the size of the vesicles (see Results section). Niosomes formed by thin film hydration were stored at room temperature and were extruded 15x through a 200 nm polycarbonate filter (Avestin). The liposome and niosome compositions are presented in Table 1.

**Table 1:** Lipid composition of niosomes and liposomes used in this study.

Mixtures	Composition	Molar ratio
Unsaturated lipids + cholesterol	DOPC; DOPE; cholesterol	2:1:1
Saturated lipids + cholesterol	DPPC; DPPE; cholesterol	2:1:1
Unsaturated surfactants + cholesterol	Tween 80, Span 80; cholesterol	35:35:30
Saturated surfactants + cholesterol	Tween 60, Span 60; cholesterol	35:35:30
Unsaturated lipids, incl. anionic	DOPC; DOPE; DOPG	12:50:38

### Detergent treatment and vesicle solubilization

To 1 mL vesicles at 2 mM of lipids or surfactants, 20 or 50μL 1 or 10% Triton-X100 was added. The change in turbidity of the vesicle suspension was recorded at 540 nm on a spectrophotometer. The A_540_ of the vesicles prior to the addition of Triton X100 only was set to 1; dilution due to Triton-X100 addition was corrected for.

### Cryo-EM and dynamic light scattering (DLS)

For cryo-EM, niosomes composed of unsaturated surfactants or liposomes composed of unsaturated lipids were subjected to five freezing and thawing cycles as indicated above and extruded 15x through a 200 nm polycarbonate filter (Avestin). To examine the effect of the freezing and thawing, similar vesicles were formed with the freezing and thawing step omitted. Vesicles (10 mM of lipids or surfactants) were placed on a grid and vitrified using a vitrobot (FEI). Images were recorded on a Tecnai T20 microscope (FEI) with a Gatan cryo-stage (model 626) operating at 200 keV. Images were recorded under low-dose conditions on a slow scan CCD Camera. DLS was performed using the Dynapro Nanostar apparatus, and the results were analyzed with dynamics software, version 7.

### Cobalt-calcein assay for cargo leakage and activity of antimicrobial peptides (AMPs)

This assay is based on a procedure described by[10]. Vesicles were prepared in 20 mM Na-MOPS, 0.8 mM calcein, 1 mM CoCl_2_, 90 mM NaCl, pH 7.5. Unencapsulated material was removed on a Sephadex G-75 column, eluted with 20 mM Na-MOPS, 50 mM NaCl, 10 mM EDTA, pH 7.5. Leakage of calcein was followed for up to 24 hours on a Jobin Yvon fluorescence spectrophotometer with excitation at 495 nm and emission at 515 nm (slit widths of 3 nm for excitation and emission). To achieve 100% leakage (I_max_), 0.25% (v/v) Triton X-100 was added. The percentage of calcein leakage was calculated by:

$$\%\;Leakage=\frac{\left(I_{t}-I_{0}\right)}{\left(I_{max}-I_{0}\right)}x100\%$$

where *I*_*t*_ is the intensity measured at the indicated time point and *I*_*0*_ the intensity at the start of the experiment. To determine the release of calcein via AMPs, we tested melittin (dissolved in water) and alamethicin (dissolved in methanol); the final methanol concentration was kept below 1% (v/v), and controls showed that methanol had no influence on the leakage.

### Lipid/surfactant packing

Lipid packing was determined with the probe Laurdan. This probe, dissolved in chloroform:methanol (9:1), was added to the vesicles, prepared in 20 mM Na-MOPS, 100 mM NaCl, pH 7.5. The fluorescence of the vesicles was measured on a Yvon Jobin fluorescence spectrophotometer, with excitation at 340 nm, emission at 440 and 490 nm, according to[11]. The generalized polarization (GP), a measure of lipid packing, is given by:

$$GP=\frac{I_{440}-I_{490}}{I_{440}+I_{490}}$$

where I_440_ and I_490_ are the emission intensity measured at 440 nm or 490 nm, respectively.

### H^+^ permeability

The H^+^ permeability was assessed using the pH sensitive dye pyranine. Vesicles were prepared in 20 mM Na-MOPS, 100 mM NaCl, 100 μM pyranine, pH 7.5; measurements were done in the absence and presence of the sodium ionophore ETH-157. To remove unencapsulated pyranine, the vesicles were eluted over a Sephadex G-75 column with 20 mM Na-MOPS, 100 mM NaCl, pH 7.5. The fluorescence was measured at 510 nm, with excitation at 400 and 450 nm and slit widths of 5 nm for excitation and emission. The measurements were done at an initial pH of 7.5 on the inside and outside, after which the pH on the outside was lowered to approximately 6.3 or 7.0 through addition of 10 or 4 mM HCl (final concentration). The pH on the inside of the vesicles was followed for 10 minutes. As a measure of pH, the logarithm of the fluorescence intensity at 450 nm divided by the fluorescence intensity at 400 nm was taken[12]; the value decreases with decreasing pH. The emission intensities at excitation wavelengths of 450 and 400 nm were converted to pH values by using standard curves of F450/F400 versus pH for pyranine in 20 mM Na-MOPS, 100 mM NaCl, entrapped in protonophore-permeabilized vesicles.

### Mechanical stability and osmotic effects

The effect of osmotic upshift on vesicles was studied by recording the calcein fluorescence. Vesicles were prepared in 20 mM Na-MOPS, 5 mM calcein, 100 mM NaCl, pH 7.5. This concentration of calcein is at the low end of self-quenching[13]. Calcein fluorescence (quenching) was measured as described above.

### Stopped-flow measurements

For fast changes in calcein fluorescence after an osmotic upshift, a stopped-flow apparatus (SX20, Applied Photophysics Lim.) was used. To impose the osmotic upshift, KCl or glycerol was loaded in one syringe and the liposome or niosome solution in the other syringe, and both were rapidly mixed (1:1 mixing ratio with 2 ms dead time) and then injected into the optical cell (20 μl volume and 2 mm path length). The band pass of the monochromator was set to 0.5 nm. Calcein was excited at 495 nm. The emitted light was filtered by a Schott long-pass filter (cut-off wavelength at 515 nm) and detected by a photomultiplier tube (Hamamatsu R6095) with 10 μs time resolution. The voltage of the photomultiplier was automatically selected and kept constant during each set of experiments. The fluorescence intensity kinetics after the osmotic upshift was recorded with logarithmically spaced time points to better resolve faster processes. For noise reduction, 5 acquisitions were performed for each experimental condition. The raw data were processed in Matlab (R2015b, MathWorks Inc.) for further analysis. The 5 curves obtained in every experimental condition were averaged and the resulting kinetic curves F(t) were normalized to one at time zero (F(t)/F(0)), i.e. the mixer dead time (t _0_ = 2 ms).

### Reconstitution of arginine/ornithine antiporter ArcD2

The expression and purification of ArcD2 will be described elsewhere (Pols *et al*., manuscript in preparation). Purified ArcD2 was reconstituted into vesicles composed of unsaturated phospholipids, niosomes composed of unsaturated surfactants and niosomes composed of saturated surfactants. Vesicle compositions are given in Table 1. Reconstitution was performed as described previously[14], using a protein to lipid (w/w) ratio of 1:400. The vesicles were prepared in 50 mM KPi, pH 7.0 containing 200 μM L-ornithine.

### Transport assays

After 15x extrusion through a 200 nm polycarbonate filter, the vesicles were diluted 4000x into 50 mM KPi, pH 7.0 without ornithine and collected by centrifugation (25 min, 444.000 x g (R_max_), 4°C). Vesicles were resuspended in 50 mM KPi, pH 7.0 to a final concentration of 1 μg ArcD2/μl. To start the uptake assay, 2 μg of reconstituted ArcD2 was added to 100 μl of 50 mM KPi, pH 7.0 with 25 μM radiolabeled L-[^14^C(U)]-arginine (specific activity of 20 mCi/mmol), while stirring at 30°C. As a negative control, 200 μM of ornithine was included in the uptake buffer. At given time points, reactions were stopped by addition of 2 ml of quenching buffer (ice cold 50 mM KPi, pH 7.0) and filtration over a 0.45 μm pore size nitrocellulose filter (GE-Healthcare). Filters were washed with an additional 2 ml of quenching buffer and dissolved in 2 ml of Ultima Gold MV scintillation solution (PerkinElmer). After vortexing, radioactivity was determined by liquid scintillation counting in a Tri-Carb 2800TR liquid scintillation analyzer (PerkinElmer).

### Miscellaneous

Purified ArcD2 fractions and proteovesicles were separated on a 12.5% SDS-PAGE gel, after which Western blotting and gel imaging was done as previously described[15]. All results presented in the manuscript are based on replicate experiments that have been carried out at least twice, and each experiment typically constitutes of several technical replicates.

## Results

### Niosomes composed of unsaturated surfactants shrink due to freezing and thawing

**Fig 2:**
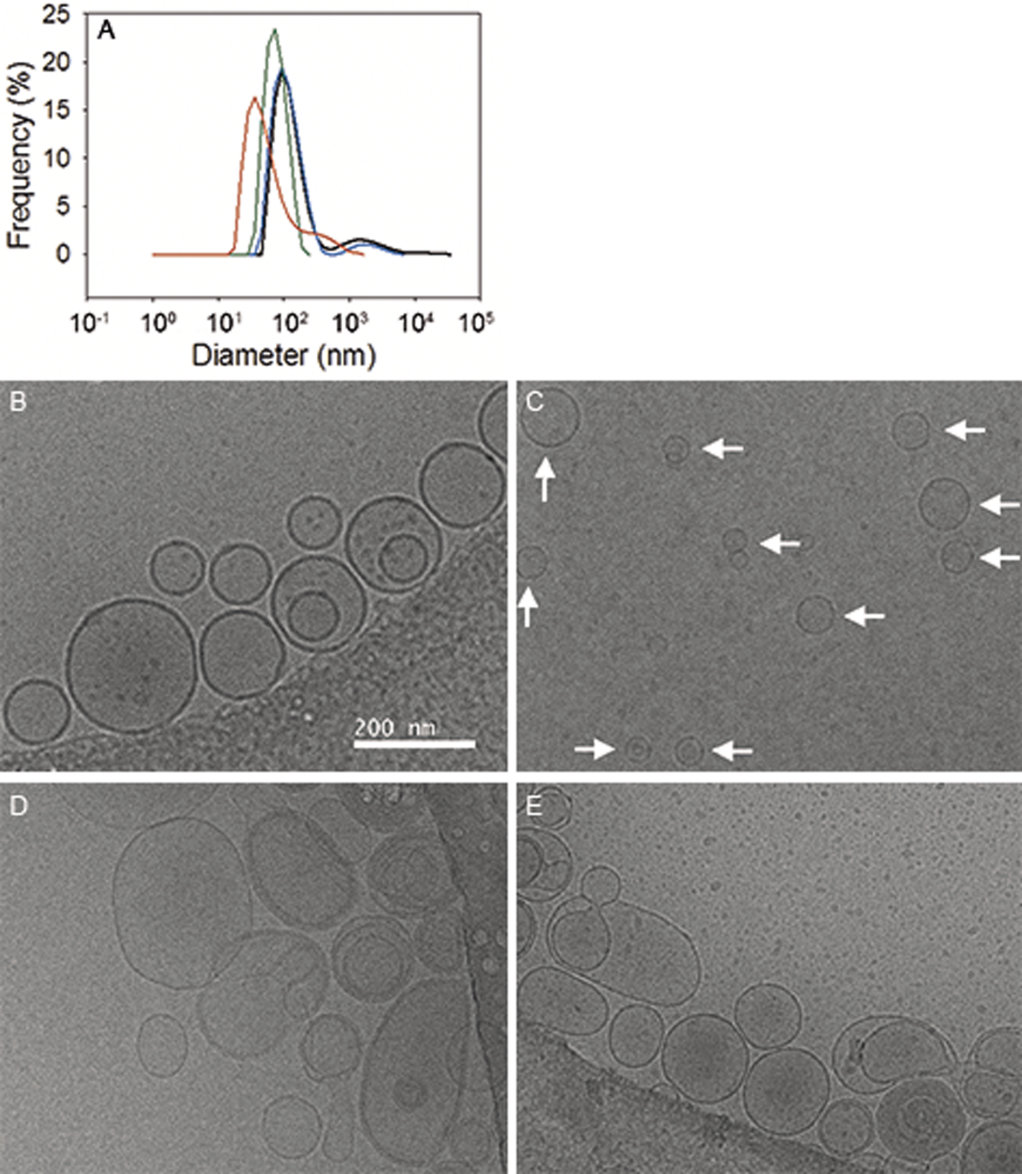
Filter-extruded niosomes decrease in size upon freezing and thawing. A: Size of vesicles composed of unsaturated surfactants plus cholesterol (green: without freezing and thawing; red: with freezing and thawing), and vesicles composed of unsaturated lipids plus cholesterol (black: without freezing and thawing; blue: with freezing and thawing), measured by dynamic light scattering. Prior to the analysis the vesicles were extruded 15 times through a 200 nm polycarbonate filter. B-C: Cryo-EM pictures of niosomes composed of unsaturated surfactants plus cholesterol without (B) and with five freeze and thaw cycles (C). Niosomes appear smaller due to the freezing and thawing steps. As guidance, all niosomes are indicated with a red arrow in the right picture. In contrast, cryo-EM pictures of liposomes composed of unsaturated lipids plus cholesterol without (D) and with five freeze and thaw cycles (E) appear similar in size but the degree of multilamellarity decreases by the freezing-thawing and subsequent extrusion step.

Initial experiments were performed with niosomes composed of unsaturated surfactants and formed using a procedure to prepare liposomes as indicated in the methods section, employing freezing and thawing to swell the vesicles. However, this procedure yielded niosomes with a very low encapsulation efficiency compared to niosomes not subjected to freeze-thaw cycles (data in S1 Fig). These observations can be explained by a decrease in vesicle size or a high permeability of the vesicles that had undergone the freeze-thaw cycles. Cryo-EM and DLS show that niosomes formed with the freeze-thaw procedure were smaller than niosomes formed without this step (Fig 2A–2C). In subsequent experiments niosomes were prepared without freezing and thawing as described in the Methods section. For liposomes composed of unsaturated lipids, the freeze-thaw step did not affect vesicle size (Fig 2A, 2D–2E).

### Niosomes and liposomes are stable for more than 24 h

**Fig 3:**
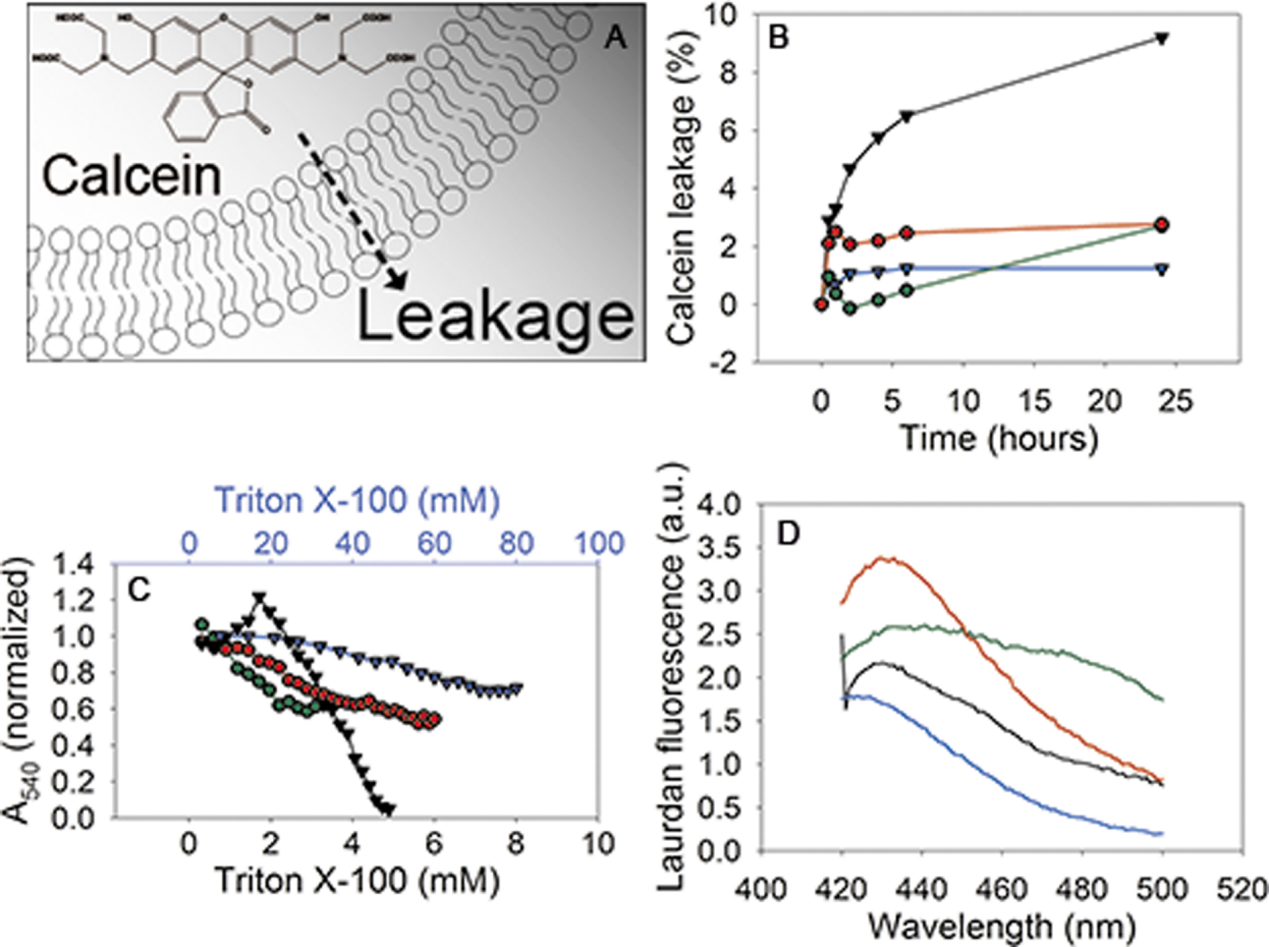
Membrane stability and packing depend on lipid and surfactant composition. A: Graphical representation of calcein leakage across membrane. B: Leakage of calcein from liposomes and niosomes. Green lines: niosomes composed of unsaturated surfactants plus cholesterol; red lines: niosomes composed of saturated surfactants plus cholesterol; black lines: liposomes composed of unsaturated lipids plus cholesterol; blue lines: liposomes composed of saturated lipids plus cholesterol. Representative traces of one out of two experiment are shown. C: Stability of niosomes and liposomes in the presence of Triton X-100. The data are corrected for dilution; line/symbol color as in B. The upper x-axis is for the liposomes composed of saturated lipids plus cholesterol. Representative traces of one out of two experiment are shown. D: Membrane packing of niosomes and liposomes, measured using the environment-sensitive dye laurdan; line/symbol color as in B. The average of two independent experiments is shown. The spectra of the corresponding vesicles without laurdan were used to correct for background signal.

We monitored the leakage of the water-soluble dye calcein from niosomes and liposomes, prepared from saturated and unsaturated amphiphiles. The fluorescence of calcein is quenched by cobalt, but upon leakage of calcein into the outside medium containing EDTA, the quenching is abolished[10]. All vesicles were stable over a 24 hour time period, as indicated by a leakage smaller than 10% (Fig 3B). The release of calcein from liposomes composed of saturated lipids could not be quantified because these vesicles are resistant to solubilization by Triton X-100, but clearly the release was minimal. In order to obtain the values in Fig 3B, the maximum fluorescence of unsaturated liposomes was taken, assuming equal encapsulation efficiency. Thus, both niosomes and liposomes retain molecules like calcein for at least one day, even when the membranes are composed of mostly unsaturated amphiphiles.

Detergent stability of liposomes composed of DPPC, DPPE and cholesterol was confirmed by turbidity measurements. With increasing detergent concentration, the vesicles ultimately become fully solubilized and the turbidity (A_540_) decreases to almost zero as seen for liposomes composed of unsaturated phospholipids, niosomes composed of unsaturated surfactants plus cholesterol, and niosomes composed of saturated surfactants plus cholesterol (Fig 3C). However, the behavior is different for niosomes and liposomes: with niosomes the A_540_ gradually decreases upon addition of Triton X-100, whereas liposomes composed of unsaturated phospholipids show a characteristic maximum in A_540_; the point at which the membrane is saturated with detergents[16–18], at higher detergent concentration the vesicles become solubilized. Liposomes composed of saturated lipids are much less affected by Triton X-100 and only a 20% decrease in A_540_ is observed with 80 mM Triton X-100 (Fig 3C, upper x-axis).

### The membrane environment of niosomes probed by generalized polarization

The membrane order was probed with the environment sensitive dye laurdan. Depending on the polarity of the environment, the maximum emission wavelength varies from 440 (apolar) to 490 nm (polar). The ratio of fluorescence intensity at 440 and 490 nm was calculated and is presented as Generalized Polarization (GP)[11]. A GP value of -1 indicates highly disordered and +1 corresponds to a highly ordered membrane. We found values of 0.1 ± 0.007 and 0.5 ± 0.09 for niosomes composed of unsaturated surfactants and saturated surfactants, respectively (Fig 3D). Liposomal membranes composed of unsaturated lipids had a GP of 0.3 ± 0.1 and membranes with saturated lipids had a GP of 0.6 ± 0.05. The packing of membranes composed of surfactants is thus somewhat less tight than that of phospholipid membranes, both in case of saturated and unsaturated membrane components.

### Ion permeability of niosomes and liposomes

**Fig 4:**
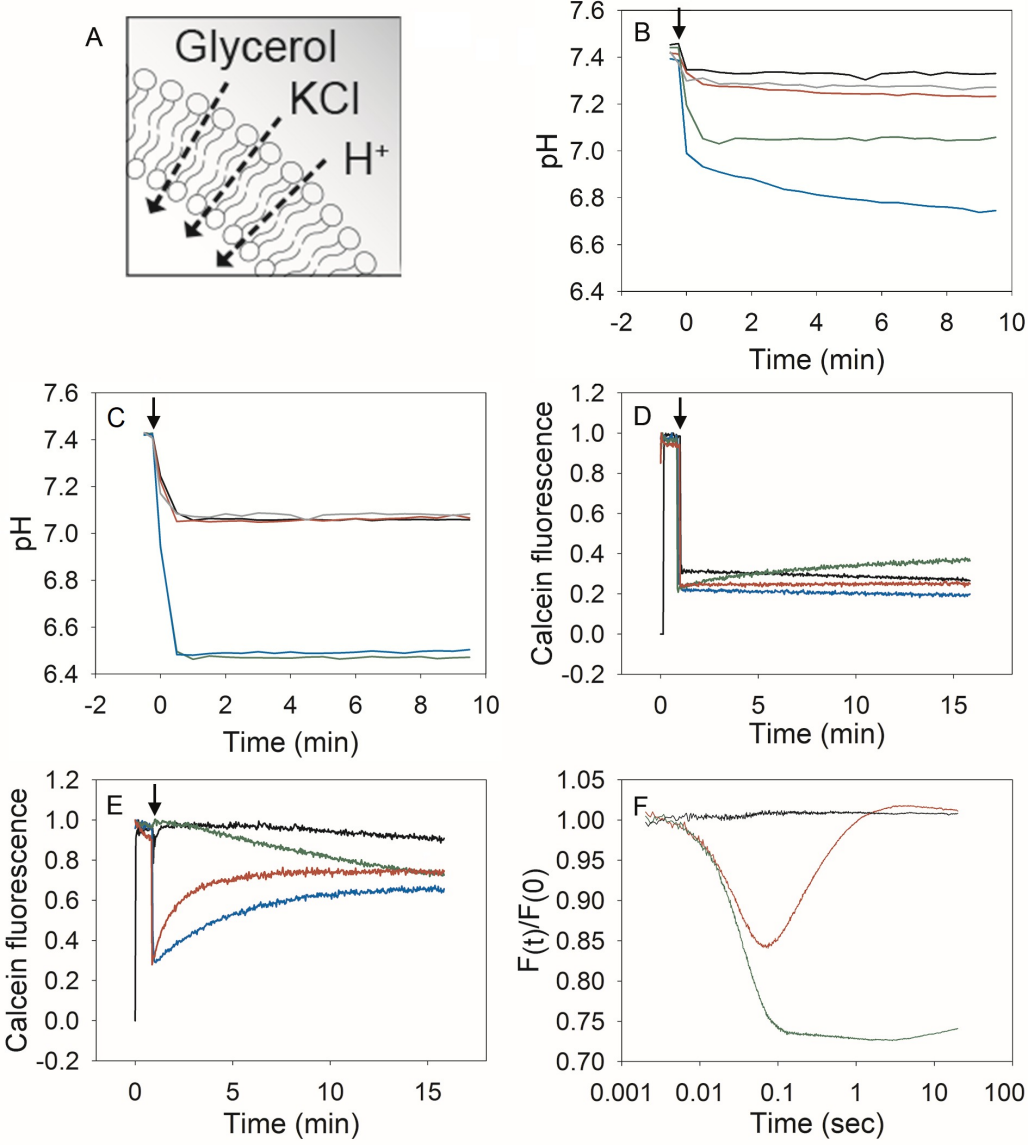
Membrane permeability of niosomes and liposomes. A: Graphical representation of the ion permeability of the vesicles. B: Proton permeability measured by fluorescence of the pH-sensitive dye pyranine in liposomes composed of unsaturated lipids plus cholesterol in the presence and absence of the sodium ionophore ETH-157. ETH-157 (5 μM, final concentration) or ethanol (0.1% v/v) were present from the start of the experiment. At time point 0 (indicated by an arrow), the medium pH was decreased from 7.5 to 6.3 by the addition of 10 mM HCl (large pulse) or from 7.5 to 7.0 by the addition of 4 mM HCl (small pulse). Black line: ethanol, small pulse; red line: ETH-157, small pulse; green line: ethanol, large pulse; blue line: ETH-157, large pulse. For comparison, liposomes composed of saturated lipids plus cholesterol subjected to a large HCl pulse (in the absence of ETH-157) are shown in grey. Average values of two experiments are shown. C: Proton permeability of niosomes composed of unsaturated surfactants plus cholesterol in the absence (0.1% v/v ethanol) or presence of the sodium ionophore ETH-157 (5 μM, final concentration). At time point 0 (indicated by an arrow), the medium pH was decreased from 7.5 to 6.3 by the addition of 10 mM HCl (large pulse) or from 7.5 to 7.0 by the addition of 4 mM HCl (small pulse). Black line: ethanol, small pulse; red line: ETH-157, small pulse; green line: ethanol, large pulse; blue line: ETH-157, large pulse. Niosomes composed of saturated surfactants plus cholesterol subjected to a large HCl pulse (in the absence of ETH-157) are shown in grey. Average values of two independent experiments are shown. D: KCl permeability of liposomes and niosomes filled with the fluorescent dye calcein (5 mM) after osmotic upshift by KCl. The arrow at 50s indicates the moment 0.4 M KCl (final concentration) was added. Green lines: niosomes composed of unsaturated surfactants plus cholesterol; red lines: niosomes composed of saturated surfactants plus cholesterol; black lines: liposomes composed of unsaturated lipids plus cholesterol; blue lines: liposomes composed of saturated lipids plus cholesterol. Representative traces of one out of three independent experiments are shown. E: Stability of liposomes and niosomes filled with the fluorescent dye calcein (5 mM) after osmotic upshift by glycerol. At 50s (indicated by a black arrow), 0.667 M glycerol was added (osmolarity comparable to that of 0.4M KCl); line color as indicated under B. Representative traces of one out of two independent experiments are shown. F: Stopped-flow measurements of the effects of osmotic upshift elicited by glycerol (red line) or KCl (green line) in niosomes composed of unsaturated surfactants plus cholesterol. Buffer (black line) is shown as a control. Representative traces of one out of two independent experiments are shown.

We compared the permeability of niosomes and liposomes for protons and compensating ion(s) by monitoring the internal pH upon acidification of the external medium (Fig 4B and 4C). In all cases, we observe a fast drop in signal that is partly attributed to residual pyranine, sticking to the surface of membranes containing zwitterionic amphiphiles, as reported by others previously [19]. The slower kinetic component takes place over a long period of time as the membranes are relatively impermeable for protons. In liposomes with unsaturated lipids and cholesterol, the internal pH dropped from 7.5 to 7.1 when the external pH was lowered from 7.5 to 6.3 (Fig 4B, green line). To determine if the build up of a membrane diffusion potential limited the proton permeability, we performed experiments in the presence of the sodium ionophore ETH-157. ETH-157 enhanced the pH decrease when the outside pH of liposomes was lowered from 7.5 to 6.3 (Fig 4B, blue line), indicating that indeed the proton permeability is limited by counterion flux and thus a membrane potential (inside positive) is formed by the large pH jump. The effect of ETH-157 was small when the pH was lowered from 7.5 to 7.0 (Fig 4, black and red lines), which is in accordance with previous observations that with small gradients diffusion potentials are negligible[20].

In niosomes with unsaturated surfactants (Span 80, Tween 80) and cholesterol, a rapid decrease in internal pH was observed in the absence and presence of ETH-157. Thus irrespective of whether a large (pH_out_ 7.5 to 6.3) or small (pH_out_ from 7.5 to 7.0) pH shift was imposed the internal and external pH equilibrated in less than one min, and it was not necessary to increase the permeability for the balancing ion (here, Na^+^ via ETH-157). Thus, niosomes composed of unsaturated surfactants and liposomes composed of unsaturated phospholipids are similarly permeable for protons but the rate of permeation of the compensating ion is higher in the niosomes (Fig 4C). In niosomes with saturated surfactants (Span 60, Tween 60) and cholesterol, the internal pH stabilized at ~7.1 when pH_out_ was lowered from 7.5 to 6.3 (Fig 4B). The internal pH of liposomes composed of saturated lipids (DPPC, DPPE and cholesterol), leveled off to a value of ~7.3 over a period of 10 min (Fig 4B).

### Mechanical stability and osmotic stress

Next, we determined the mechanical stability of the vesicles by imposing an osmotic upshift, using membrane impermeable (KCl; Fig 4D) and membrane permeable (glycerol; Fig 4E) osmolytes. After an osmotic upshift by the addition of KCl or glycerol to the outside medium, the vesicles release water and deform (shrink). This leads to a higher level of self-quenching of calcein and decreased fluorescence intensity. If the membrane is relatively permeable for the osmolyte (*e*.*g*. glycerol) then the vesicles will return to their normal shape and volume within seconds or minutes, which is observed as relief of self-quenching. With relatively impermeable osmolytes (*e*.*g*. KCl) the vesicles maintain their shrunken state up to hours. We found that all the vesicles remain intact (stable) after an osmotic upshift with KCl, except for the niosomes composed of Tween 80, Span 80 plus cholesterol. Here, we observed a small relief of the quenching, indicating that potassium or/and chloride ions are diffusing slowly across the membrane with unsaturated surfactants.

We found that the relief of quenching upon addition of glycerol takes minutes in niosomes prepared of Tween 60, Span 60 plus cholesterol; similar effects are observed in liposomes composed of saturated lipids plus cholesterol. In contrast, self-quenching is not observed in niosomes composed of Tween 80, Span 80 plus cholesterol, and it is barely visible in liposomes composed of unsaturated lipids plus cholesterol. We hypothesized that in niosomes composed of unsaturated amphiphiles the membranes are so permeable for glycerol that the time resolution (sec) of our measurements is insufficient to probe the transient shrinkage of the vesicles. Indeed, in stopped flow experiments where we have a time resolution better than 10 ms, we clearly observe the kinetics of glycerol permeation. In Fig 4F we show the shrinkage of the vesicles (calcein quenching) upon addition of glycerol (red line) and KCl (green line). The niosomes recover from the glycerol stress within 1 sec; with KCl the shrinkage is permanent on the sec to min timescale.

We cannot explain the gradual decrease in fluorescence in niosomes composed of unsaturated surfactants and cholesterol upon addition of glycerol (Fig 4E, green line). It must due to bleaching of calcein that is caused by the combination of glycerol and unsaturated amphiphiles or an unknown component herein. Overall, the results presented in Fig 4 indicate that both liposomes and niosomes composed of saturated amphiphiles form stable vesicles and that their membranes are relatively impermeable to ions and moderately permeable to glycerol. Niosomes composed of unsaturated amphiphiles are more permeable to ions (protons, KCl) than liposomes composed of unsaturated lipids.

### Antimicrobial peptides act on niosomes

**Fig 5:**
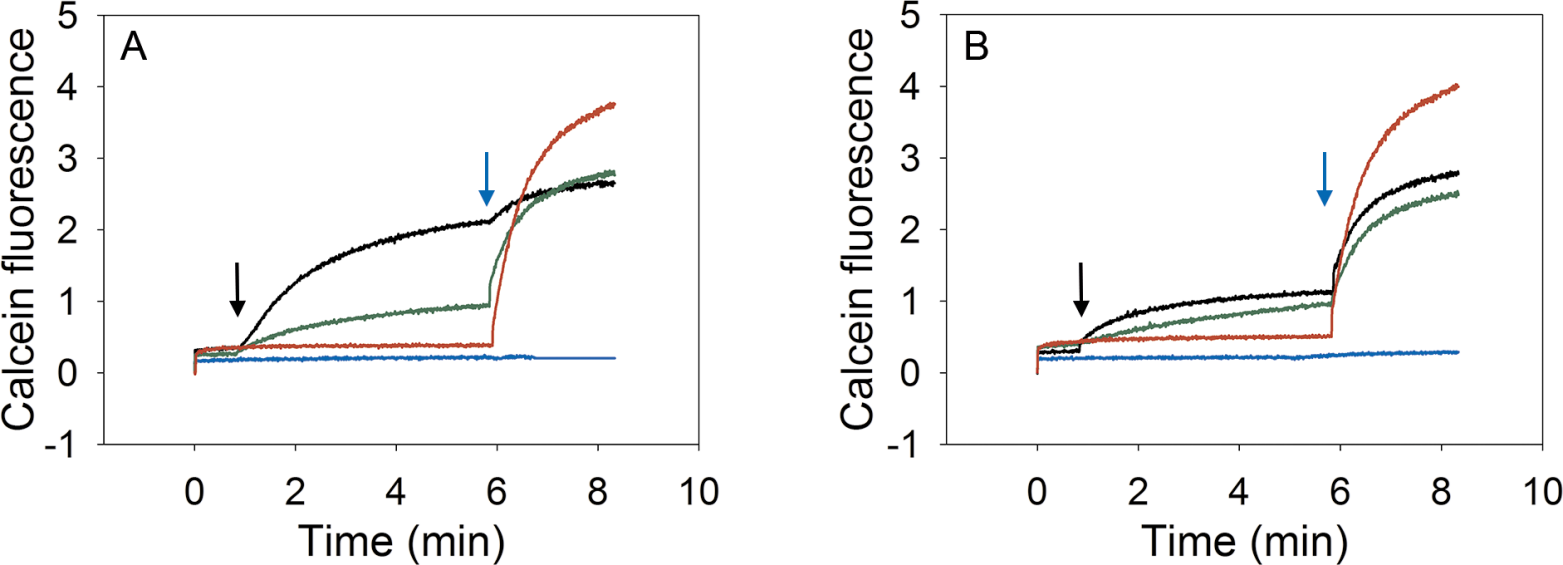
Melittin and alamethicin induce calcein leakage. A: To vesicles filled with 20 mM Na-MOPS, 1 mM CoCl_2_ and 0.8 mM calcein, 90 mM NaCl, pH 7.5, 1 μM melittin (final concentration) was added at the time of the black arrow. To obtain the maximum signal, 0.25% (v/v) Triton X-100 was added after 350 s (indicated by the blue arrow). Green lines: niosomes composed of unsaturated surfactants plus cholesterol; red lines: niosomes composed of saturated surfactants plus cholesterol; black lines: liposomes composed of unsaturated lipids plus cholesterol; blue lines: liposomes composed of saturated lipids plus cholesterol. Representative traces of one out of two independent experiments are shown. B: To vesicles as in A, 5 μM alamethicin (final concentration) was added at the time of the black arrow. To obtain the maximum signal, 0.25% Triton X-100 was added after 350 s (indicated by the blue arrow); line color as indicated under A. Representative traces of one out of two independent experiments are shown.

The membrane lipid composition can have major impact on the function and activity of antimicrobial peptides (AMPs), *e*.*g*. on the interaction of the peptides with the membrane[21–23] or the mechanism of pore formation[24]. When alamethicin or melittin were added to niosomes composed of Tween 80, Span 80 plus cholesterol, a rapid release of calcein was observed, similar to the release of calcein from liposomes composed of the unsaturated lipids DOPC, DOPE plus cholesterol (Fig 5A and 5B). Both peptides act in the micromolar range, corresponding to AMP to amphiphile ratios of roughly 1 to 20 for melittin and 1 to 4 for alamethicin. As expected, both AMPs did not affect vesicles composed of saturated amphiphiles, be it niosomes or liposomes.

### Membrane transporter activity

**Fig 6:**
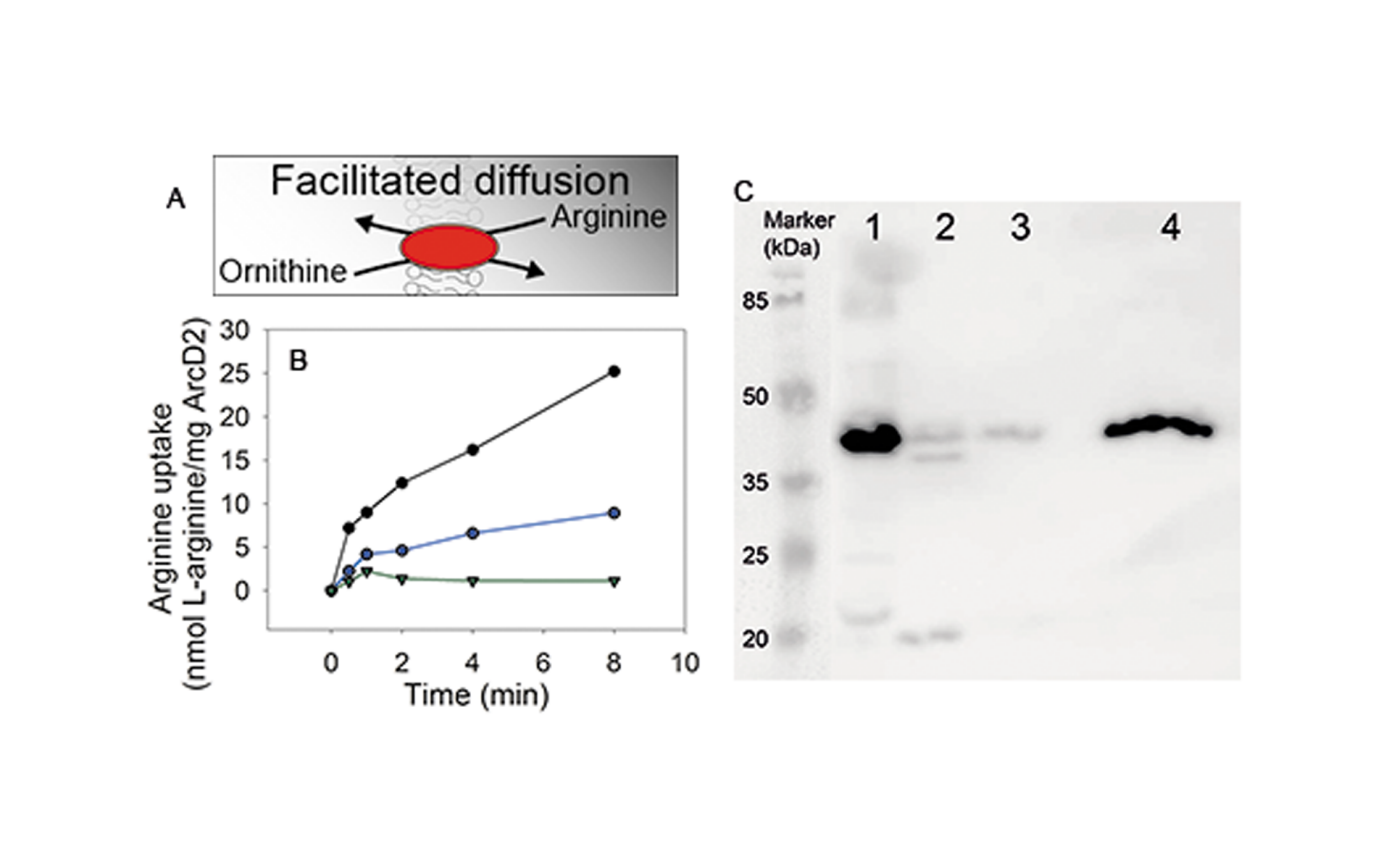
The L-arginine/L-ornithine antiporter ArcD2 is active in liposomes with anionic lipids but not in vesicles that do not contain lipids or surfactants with anionic headgroups. ArcD2 was reconstituted in liposomes and niosomes at 1 to 400 protein to lipid ratio (w/w). A: Schematic representation of the transport reaction. B: ArcD activity was measured using radiolabeled arginine. Green lines: niosomes composed of unsaturated surfactants plus cholesterol; blue lines: liposomes composed of unsaturated lipids plus cholesterol; black lines: liposomes composed of unsaturated lipids of which 38% is anionic (phosphatidylglycerol). Representative traces of one out of three independent experiments are shown. C: Incorporation of ArcD2 into vesicles was confirmed by Western blot analysis. 1: solubilized ArcD2, purified protein before reconstitution; 2: Unsaturated surfactants + cholesterol; 3: Saturated surfactants + cholesterol; 4: Unsaturated lipids + cholesterol.

Given the finding that alamethicin and melittin act functionally in niosomes, we wondered whether we could reconstitute a simple secondary transporter in membranes composed of Tween 80, Span 80 plus cholesterol. The L-arginine/L-ornithine antiporter ArcD2 does not require an electrochemical ion gradient or other source of metabolic energy for transport. ArcD2 was reconstituted in niosomes composed of unsaturated surfactants, niosomes composed of saturated surfactants and in liposomes composed of the unsaturated lipids DOPC, DOPE and cholesterol, but in all cases we did not detect any transport activity (Fig 6B). When analyzing the niosomes and liposomes on Western blot we found that ArcD2 is associated with all the vesicles, although the protein reconstitutes much better into liposomes than niosomes (Fig 6C). The molecular weight of ArcD2 is 56.7 kDa but migrates at a position of about 40 kDa, which is typical for hydrophobic proteins[25]. Contrary to the reconstitution into niosomes and liposomes composed of unsaturated, neutral lipids, antiport activity was observed in the liposomes composed of unsaturated lipids with a fraction being anionic rather than zwitterionic (Fig 6B).

## Discussion

We present a first characterization of the functional properties of niosomes composed ternary surfactant mixtures and benchmark the measurements against liposomes composed of saturated or unsaturated phosphatidylcholine-, and phosphatidylethanolamine-based lipids plus cholesterol. Most measurements were done in buffer at pH 7.5 and at room temperature. We find that niosomes leak little or no calcein (hydrophilic fluorophore with a molecular weight of 623 g/mol) over a period of 24 hours, but niosomes composed of unsaturated amphiphiles are more permeable to larger ions (KCl, compensating ions in the proton permeability measurements) than conventional lipid-based liposomes. Similar to liposomes, niosomes composed of saturated amphiphiles are more stable and less leaky when tested below the phase transition temperature (T_M_) than vesicles composed of unsaturated components; the T_M_ of Span 60 is 55°C[4, 26] and T_M_ of Span 80 is -20.3°C. The antimicrobial peptides melittin and alamethicin permeabilize both niosomes and liposomes, but we were not able to functionally reconstitute into niosomes the amino acid antiporter ArcD2. Taken together, niosomes behave in many aspects similar to phospholipid-based vesicles but they may not provide the right lipid head-group composition for functional membrane transport, that is, the requirement of many transporters for a fraction of anionic headgroups[27].

Since liposomes are widely used as vehicles for drug delivery and gene transfer, their stability and permeability properties of lipid-based vesicles have been widely studied. For instance, small unilamellar vesicles (SUVs) retain 80–95% of carboxyfluorescein (CF) in SUVs over a period of 1 hour at 37°C, depending on the type of lipids used[28]. Crommelin and Van Bommel[29] found that liposomes composed of saturated lipids with or without cholesterol had over 90% of the CF entrapped after one month of storage at 4 ^o^C. EggPC liposomes with or without cholesterol were found to leak 50% of the encapsulated calcein over 200 hours at 22°C, although this value is decreased to 23 hours at 37°C[30].

For niosomes, much less data is available on the leakage of water-soluble compounds than for liposomes. Span 60/cholesterol/dicetyl phosphate vesicles release 25% of their carboxyfluorescein compared to 45% in Span80/cholesterol/dicetylphosphate over a time period of 6h[4], indicating that niosomes containing saturated surfactants are more stable. In addition, under conditions comparable to that in our work, Span 80 vesicles released about 85% of brilliant blue after 24h[7]. These results are in line with the less than 10% leakage found by us and shows water-soluble dyes are well retained by surfactant-based membranes.

The permeability of membranes to ions and non-ionic solutes is dependent on the polarity and membrane charge, with cation permeability increasing with the fraction of anionic lipids[31]. Cationic membranes are relatively more permeable for anions (e.g. Cl^-^) than zwitterionic or anionic membranes. The permeability of zwitterionic membranes is higher for Cl^-^ anions than for Na^+^ or K^+^ cations[32, 33]. The presence of cholesterol typically decreases permeability of phosphatidyl choline membranes for Cl^-^[32]. These data are consistent with the findings we report here, but our niosomes composed of unsaturated amphiphiles are more permeable than the tested liposomes of unsaturated phospholipids.

The proton permeability of membranes is highly dependent on the head group, acyl chain length (membrane thickness) and lipid saturation dependent, and five orders of magnitude differences in permeability have been reported[34–36]. These differences are attributed to differences in pH gradient (driving force for proton permeability) and the ability of protons to move along hydrogen bonds[37]. The H^+^ permeability is several orders of magnitude higher than the permeability of similar membranes for larger cations (10^−3^ to 10^−8^ cm s^-1^ for protons[37] versus 10^−10^ to 10^−12^ cm s^-1^ for sodium[35]). The rate of proton permeability depends on the extent of the pH difference. However, a large pH gradient (> 1 pH unit) can result in a diffusion potential and thereby limit the further permeation of protons [34], which is what we see in vesicles composed of the unsaturated lipids DOPC and DOPE plus cholesterol. For niosomes, no values for ion permeability have been reported so far. Our data indicate that the H^+^ permeability in niosomes composed of saturated amphiphiles is comparable to that of the corresponding liposomes. Similarly, the H^+^ permeability in niosomes composed of unsaturated amphiphiles is similar to that of the corresponding phospholipid vesicles.

In contrast to ions, non-ionic solutes such as glycerol permeate membranes composed of unsaturated phospholipids quickly[38]. The permeability for glycerol is increased when the degree of saturation and chain length of the phospholipids decreases[39]. Cholesterol decreases the permeability off egg lecithin, POPC, DOPC plus DLPC liposomes for glucose, glycerol and rubidium ions[39, 40]. The effect of (un)saturation on permeability is confirmed here and found for niosomes and liposomes. We find that glycerol equilibrates within seconds over membranes composed of unsaturated phospholipids plus cholesterol and even faster in niosomes composed of the unsaturated surfactants Tween 80, Span 80 plus cholesterol, while it takes minutes for vesicles composed of the saturated phospholipids plus cholesterol or niosomes composed of the saturated surfactants Tween 60, Span 60 plus cholesterol.

The laurdan measurements indicate that the membrane packing of niosomes is somewhat less dense than that of liposomes. The GP range found for liposomes tested here are comparable to values reported in the literature[11, 41]. For vesicles composed of lipids that are in the gel phase, a GP value of 0.6 was found, as compared to a value of -0.2 for membrane in the liquid crystalline phase[11]. For the liquid ordered (L_o_) and liquid disordered (L_d_) phase of GUVs, prepared from stearyl sphingomyelin, DOPC and cholesterol, GP values of 0.9 and 0.2 were found, respectively[41]. Another study found GP values of 0.6 and -0.2 for similar GUVs[42]. These last two GUV studies were performed with microscopy instead of spectroscopy, so direct comparison should be taken with caution.

The membrane penetrating peptides melittin and alamethicin induced calcein leakage in niosomes and liposomes composed of unsaturated amphiphiles. Melittin is reportedly inactive on DPPC membranes below the phase transition temperature, but induces micelle formation in small unilamellar vesicles (SUVs) below the transition temperature[43]. Below the phase transition temperature, melittin binds less efficiently to phospholipids as shown by tryptophan dequenching experiments[44]. Furthermore, the more unsaturated the lipid tails are, the more effective melittin inserts into the membrane and induces leakage[45]. However, a study by Rex[46] shows that liposomes with one unsaturated bond per lipid (POPC) shows more leakage after exposure to melittin than DOPC with unsaturated bonds in both acyl chains. When cholesterol is present, DPPC membranes become even more resistant to melittin[47, 48]. The membrane-stabilizing effect of cholesterol is also observed for other lipid mixtures[49], and cholesterol even prevents melittin action by preventing the peptide to bind to the membrane[50]. Wessman and coworkers have proposed that cholesterol decreases the affinity between melittin and the membrane, but that the effective concentration of melittin in the membrane required for leakage remains the same[51]. We find that melittin acts similarly for calcein release from niosomes and liposomes.

In conclusion, niosomes exhibit physical chemical properties similar to those of liposomes, albeit that permeability for small ions/solutes is higher, which make them potentially attractive as drug carriers or delivery systems for all sorts of molecules (reviewed in[2]). Compared to liposomes, niosomes have the advantage that the components are extremely cheap compared to phospholipids, and both the lipids and non-ionic surfactants are similarly stable. A disadvantage is that the currently commercially available surfactants (Spans and Tweens) are polydisperse. Drug incorporation into niosomes has been accomplished and we show that for retention of cargo the vesicles should not be frozen and thawed. Initial animal studies have been performed with niosomes, but clinical studies have not been reported[2]. For drug administration with liposomes[52, 53], the requirements of liposomes as delivery vehicles have been formulated[54]. Many of these (size, charge and stability) are met with the niosomes described here.

## Supporting information

S1 Fig Encapsulation efficiency of the various vesicle types. Encapsulation efficiency was determined by calcein fluorescence as described by [1]. Briefly, vesicles were formed and subjected to five freeze-thaw cycles before extrusion (blue bars) or extruded without the freeze-thaw step (black bars). Vesicles were diluted 1000x and fluorescence was measured. Calcein outside the vesicles was quenched by addition of 10 μM CoCl2 (fluorescence from inside the vesicles remained). Then, the vesicles were disrupted by addition of 0.25% Triton X-100 to determine the background fluorescence. For niosomes composed of unsaturated surfactants and cholesterol (condition 1), encapsulation efficiency decreased from 0.8% to 0.3% after freeze-thaw steps. For niosomes composed of saturated surfactants and cholesterol (condition 2), freezing and thawing decreased the encapsulation efficiency from 1 to 0.7%. In liposomes, freezing and thawing affected the encapsulation efficiency to a smaller extend then in niosomes. The encapsulation efficiency decreased from 1.6 to 1.3% in liposomes composed of unsaturated lipids and cholesterol (condition 3) and 0.3 to 0.2% in liposomes composed of saturated lipids and cholesterol (condition 4).
(DOCX)
