# Supplementary material for: Niosomes, an alternative for liposomal delivery
Source: PLoS One. 2018 Apr 12;13(4):e0194179. doi: 10.1371/journal.pone.0194179 (PMC5896898; doi:10.1371/journal.pone.0194179)
Supplement: S1 Fig — Encapsulation efficiency was determined by calcein fluorescence as described by [1]. Briefly, vesicles were formed and subjected to five freeze-thaw cycles before extrusion (blue bars) or extruded without the freeze-thaw step (black bars). Vesicles were diluted 1000x and fluorescence was measured. Calcein outside the vesicles was quenched by addition of 10 μM CoCl2 (fluorescence from inside the vesicles remained). Then, the vesicles were disrupted by addition of 0.25% Triton X-100 to determine the background fluorescence. For niosomes composed of unsaturated surfactants and cholesterol (condition 1), encapsulation efficiency decreased from 0.8% to 0.3% after freeze-thaw steps. For niosomes composed of saturated surfactants and cholesterol (condition 2), freezing and thawing decreased the encapsulation efficiency from 1 to 0.7%. In liposomes, freezing and thawing affected the encapsulation efficiency to a smaller extend then in niosomes. The encapsulation efficiency decreased from 1.6 to 1.3% in liposomes composed of unsaturated lipids and cholesterol (condition 3) and 0.3 to 0.2% in liposomes composed of saturated lipids and cholesterol (condition 4). (DOCX) [file pone.0194179.s001.docx]

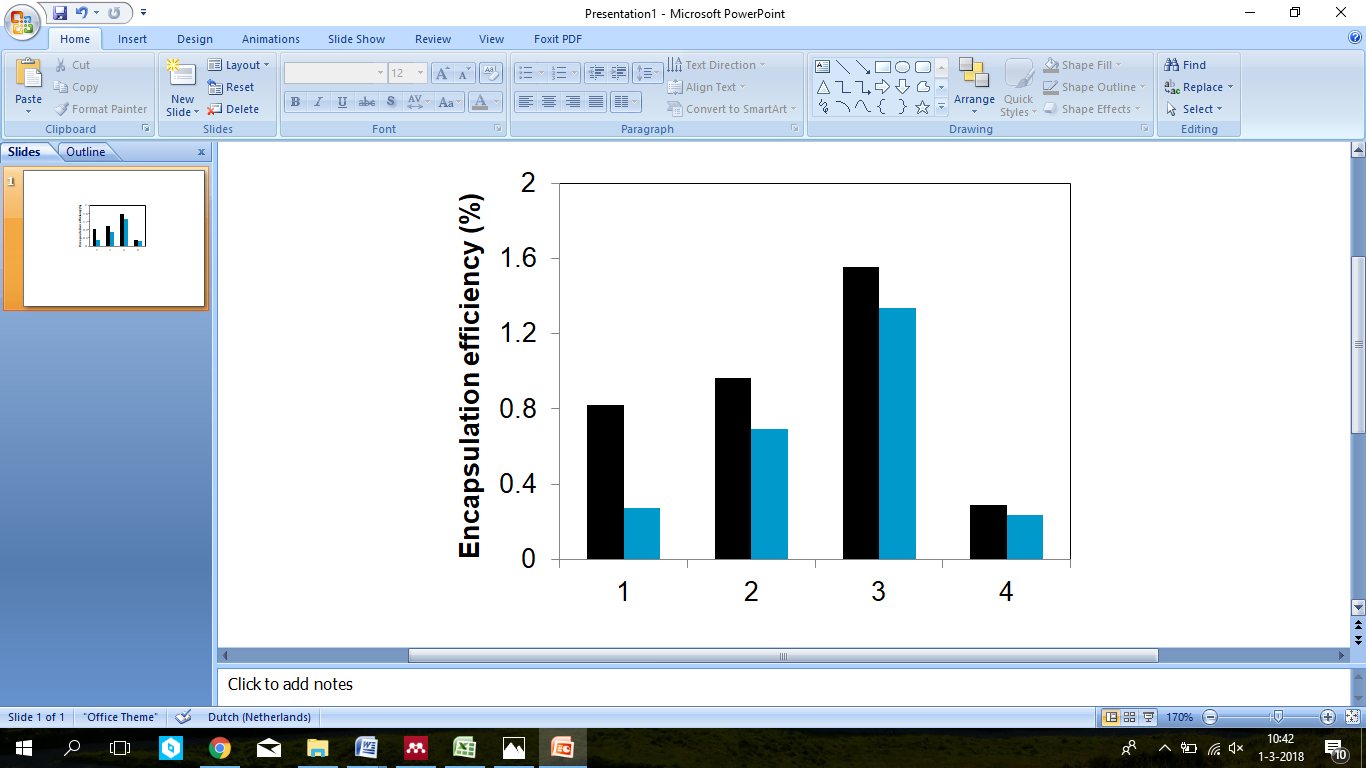


1. Oku, N., Kendall, D. A., & MacDonald, R. C. (1982). A simple procedure for the determination of the trapped volume of liposomes. *BBA-Biomembranes*, *691*(2), 332–340.
